# Aflatoxin B_1_ inhibits the type 1 interferon response pathway via STAT1 suggesting another mechanism of hepatocellular carcinoma

**DOI:** 10.1186/s13027-017-0127-8

**Published:** 2017-03-20

**Authors:** Patrick W. Narkwa, David J. Blackbourn, Mohamed Mutocheluh

**Affiliations:** 1 Department of Clinical Microbiology, School of Medical Sciences, Kwame Nkrumah University of Science and Technology, Kumasi, Ghana; 2 Department of Microbial and Cellular Sciences, School of Biosciences and Medicine, University of Surrey, Surrey, GU2 7XH UK

**Keywords:** Aflatoxin B_1_, Hepatocellular carcinoma, STAT1, Type I interferon pathway, HepG2 cells, JAK1, ISRE

## Abstract

**Background:**

Aflatoxin B_1_ (AFB_1_) contamination of food is very high in most sub-Saharan African countries. AFB_1_ is known to cause hepatocellular carcinoma (HCC) by inducing mutation in the tumour suppressor gene TP53. The number of new HCC cases is high in West Africa with an accompanying high mortality. The type I interferon (IFN) pathway of the innate immune system limits viral infections and exerts its anti-cancer property by up-regulating tumour suppressor activities and pro-apoptotic pathways. Indeed, IFN-α is reported to show significant protective effects against hepatic fibrogenesis and carcinogenesis. However, the mechanism behind AFB_1_ deregulation of the type I interferon (IFN) signalling pathway, with consequent HCC is largely unknown. This current study seeks to test the hypothesis that AFB_1_ inhibits the type I IFN response by directly interfering with key signalling proteins and thus increase the risk of HCC in humans.

**Methods:**

We evaluated the effects of AFB_1_ on the type I IFN signalling pathway using IFN stimulated response element (ISRE)-based luciferase reporter gene assay. In addition, the effects of AFB_1_ on the transcript levels of *JAK1*, *STAT1* and *OAS3* were assessed by real-time quantitative polymerase chain reaction (RT-qPCR) and confirmed by immunoblot assay.

**Results:**

Our results indicated that AFB_1_ inhibited the type I IFN signalling pathway in human hepatoma cell line HepG2 cells by suppressing the transcript levels of *JAK1*, *STAT1* and *OAS3*. AFB_1_ also decreased the accumulation of STAT1 protein.

**Conclusion:**

The inhibition of the type I IFN anti-cancer response pathway by AFB_1_ suggest a novel mechanism by which AFB_1_ may induce hepatocellular carcinoma in humans.

## Background

The innate immune response is activated within few hours upon exposure of the human system to infectious agents and other toxic chemical compounds such as mycotoxins and works to protect the individual against the harmful effects of the chemical agents and the disease causing microorganisms. One component of the innate immune system that plays a key role in the first line of defence in eliminating pathogens and tumour cells is the IFN system. The type I IFNs for example in addition to their antiviral properties have been employed in the treatment of certain cancers such as Hairy cell Leukemia, AIDS-related Kaposi’s sarcoma and other malignancies [1]. It has been reported that treatment of cells with IFN leads to the activation of the tumour suppressor gene p53 which plays a central role in the apoptosis of some tumour cells [1]. Indeed, Aziz and co-workers showed in their study that IFN-α has a significant protective effects against hepatic fibrogenesis and carcinogenesis [2]. Therefore any substance being component of pathogen or chemical produced by microorganisms which tend to inhibit or suppress the type I IFN will weaken the innate immune system and predispose individuals to infections and cancers.

AFB_1,_ a lethal mycotoxin produced by *Aspergillus flavus* and *Aspergillus parasiticus* is a potent hepatocarcinogen in humans [3, 4] and in view of that it has been classified as group 1 human carcinogen by the International Agency for Research on Cancer (IARC) [5]. AFB_1_ contamination of diet coupled with subsequent prolonged heavy exposure is a major risk factor for the development of HCC. Food meant for human and animal consumption have been reported to contain high levels of aflatoxins in some West African countries such as Ghana, Togo, Nigeria and Benin [6–8] largely due to sub-optimal farming practices, high humidity and poor storage conditions. For example in Ghana 83.3% of weanimix, food prepared locally from maize and groundnut for children have been reported to have aflatoxin levels higher than the national acceptable levels of 15 ppb [9]. Cereal based foods are staple in Ghana in particular and sub-Saharan Africa in general, it means many more people are exposed to high levels of aflatoxins and thus increasing their risk of HCC.

The incidence of HCC in the West African sub region is high with an annual death rate of about 200,000 [10]. In fact West Africa is ranked second, aside Eastern Asia as region affected most with HCC [10]. In West Africa, the death rate of HCC is almost equal to its incidence with most HCC sufferers dying within weeks of their diagnosis indicating the aggressive and dangerous nature of HCC [10, 11]. In addition to AFB_1_, other risk factors that contribute to HCC include chronic HBV/HCV infection and heavy alcohol consumption. Information available indicates that the risk of HCC developing is amplified through the synergistic effects of aflatoxin ingestion and HBV infection. The risk of HCC in people with chronic HBV infection and also exposed to aflatoxin is up to 30 times greater than in individual exposed to either of the two factors only [12–14]. These two risk factors (aflatoxin and HBV) are common in underdeveloped countries of the world including Ghana [6, 15] suggesting that the risk of HCC is likely to be high in Ghana.

The role of AFB_1_ in the pathogenesis of HCC, via mutation in the tumour suppressor gene p53 has been well established. However, information on how AFB_1_ could deregulate other anti-cancer pathways such as the type I IFN signalling pathway as a way of causing HCC is very limited. This study was carried out to test the hypothesis that AFB_1_ inhibits the type I IFN response pathway thus contributing to the pathogenesis of HCC. Results from this study could influence future therapeutic intervention for AFB_1_-induced HCC and also broaden our knowledge of the role of AFB_1_ in HCC immunobiology.

## Methods

### Reagents and chemicals

The AFB_1_ used in the study was purchased from Sigma-Aldrich, USA (cat no A6636) and dissolved in dimethyl sulfoxide (DMSO) to a stock concentration of 3200 μM. The AFB_1_ stock solution was divided into aliquots, wrapped in aluminium foil and stored frozen at −20 °C until used. The AFB_1_ stock solution was diluted to the desired concentration in normal growth medium when necessary. The foetal bovine serum (FBS) was purchased from Sigma-Aldrich, USA. Dulbecco’s Modified Eagles Medium (DMEM) (high glucose, L-glutamine, sodium pyruvate and 25 mM HEPES) was purchased from Science Cell. Minimum essential medium (MEM) non-essential amino acids was purchased from Sigma Aldrich, USA while penicillin-streptomycin was purchased from Gibco by Invitrogen, UK. Lipofectamine 2000 was purchased from Gibco by Life Technologies, UK (cat no 11668-019). The human recombinant interferon-alpha 2 (rIFN-α2) was purchased from PBL interferon source (cat no 11115-1). The stock solution of the human recombinant interferon-alpha 2 was diluted to working concentration using phosphate buffered saline containing 0.1% bovine serum albumen as a diluent. The STAT1 (cat no PA5-34504) and GAPDH (cat no QE 212271) primary antibodies were purchased from Thermo Scientific, USA. The secondary antibody conjugated to horse-radish peroxidase (cat no 31430) was purchased from Thermo Scientific, USA.

### Cell culture

The cells used in this study were kindly donated by Professor David J. Blackbourn of the University of Surrey, UK. The cell lines used were human hepatoma cell line HepG2 (ECACC 85011430) and mouse fibroblast cell line L929 (NCTC) (ECACC 85103115). The HepG2 and the L929 cells were grown in DMEM high glucose containing L-glutamine, sodium pyruvate and HEPES supplemented with 10% v/v heat inactivated FBS, 1% v/v MEM non-essential amino acids, 100 IU/ml of penicillin and 100 μg/ml of streptomycin. The cultures were maintained at 37 °C in 5% carbon dioxide (CO_2_) under humidified condition.

### Cytotoxicity assay

HepG2 cells were grown to about 60% confluence and then treated with increasing concentrations of AFB_1_ (0–3200 μM). Twenty four hours later, the AFB_1_ containing medium was removed and fresh medium without AFB_1_ was added. The cytotoxic effects was evaluated by an MTS based assay using Cell Titre 96 AQueous One Solution reagent (Promega, USA, cat no G358C) following the manufacturer’s instruction.

### Transient transfections and luciferase assays in HepG2 cells

Dual luciferase assays were performed according to our previous study [16]. Briefly, the cells were grown in duplicate wells of the 96-well plate until they reached about 80% confluence. The plasmids used in this study were kind gifts from Professor David J. Blackbourn (University of Surrey, UK). The DNA, pISRE-luc used in the study was extracted from *E.coli* strain HD5α using EndoFree Maxi Prep Kit (Qiagen, USA) following the manufacturer’s instruction. The pISRE-luc expresses the firefly luciferase protein while pRLSV40 plasmid expresses the *Renilla* luciferase. The *Renilla* was included as internal control to which the pISRE-luc activity was normalized. A transfection mixture was prepared by diluting the plasmids DNA (pISRE-luc 500 ng: pRLSV40 1 ng) in serum and antibiotic free media and incubated at room temperature for 5 min. In addition, the Lipofectamine 2000 was also diluted in serum and antibiotic free media. After 5 min of incubation, the diluted DNA and Lipofectamine 2000 were mixed and incubated at room temperature for 20 min and then added to the designated wells and incubated at 37 °C in 5% CO_2_ under humidified condition for 24 h.

For the experiment that involved the determination of the minimum concentration of rIFN-α that would induce the maximum activity of IFN-α-inducible pISRE-luc activity, the transfected cells were stimulated with increasing concentrations of rIFN-α (100-400 IU/ml). Twenty four hours later, luciferase assays were performed using the dual luciferase reporter assay system (Promega, USA, cat no E1960) following the manufacturer’s protocol. After preparing the cell lysates, 20 μl of the aliquot was employed for luminescence measurement using Berthold Orion luminometer (Berthold Detection Systems, Germany).

For the experiment that involved the determination of the effects of AFB_1_ on the type 1 IFN signalling pathway, the transfected cells were stimulated with rIFN-α (400 IU/ml) and simultaneously treated with increasing concentrations of AFB_1_ (0.8–32 μM). Twenty four hours later, dual luciferase reporter gene assay was performed as described above.

### Reverse transcriptase-quantitative polymerase chain reaction (RT-qPCR)

The cultured HepG2 cells were treated with AFB_1_ and simultaneously stimulated with the rIFN-α for 24 h as described above. Total RNA was then extracted using Gene JET RNA purification kit (Thermo Scientific, Germany) following the instructions of the manufacturer. The quantity and the purity of the total RNA was verified by spectroscopy (Nano Drop 1000, Thermo Scientific). The purity was later confirmed by 1% agarose gel electrophoresis using ethidium bromide as stain.

Prior to the cDNA synthesis, any traces of genomic DNA present in the total RNA was removed by treating the total RNAs with double stranded (ds) DNase (Thermo Scientific, Germany) at 37 °C for 2 min followed by maintenance of the mixture on ice. The total RNAs were converted to cDNA using Moloney Murine Leukemia virus (M-Mul V) reverse transcriptase, oligo (dT) and random hexamer primers in a final reaction volume of 20 μl. The mixture was first incubated for 10 min at room temperature followed by further 15 min of incubation at 50 °C. The entire cDNA synthesis reaction was stopped by heating the mixture at 85 °C for 5 min. The cDNA was stored frozen at −80 °C until used in the qPCR.

JAK1, STAT1 and OAS3 target genes were amplified using the Maxima Probe/Rox qPCR master mix (Thermo Scientific, Germany). The primers and probes used were designed and synthesized by Biomers, Germany (Table 1).

**Table 1:** Sequences of probes and primers

Name of gene	Sequence of primers and probes	Fluorophores
*JAK1*	Probe: 5′AGCAGTCAGTGTGGCGTCATTCTCC-3′ Forward primer 5′- CAATTGGCATGGAACCAACGAC-3′ Reverse primer 5′-CAAATCATACTGTCCCTGAGCAAAC-3′	5′ FAM- 3′ BHQ-1
*STAT1*	Probe: 5′-CGCTCTGCTGTCTCCGCTTCCACTCC-3′ Forward primer: 5′GTTGCTGAATGTCACTGAACTTACC-3′ Reverse primer: 5′- AGCTGATCCAAGCAAGCATTGG-3′	5′ FAM- 3′ BHQ-1
*OAS3*	Probe 5′- AGCCTGGTGCCTGCCTTCAATGTCC-3′ Forward primer: 5′-TCCGCCTGACATCCGTAGATC-3′ Reverse primer: 5′-TCCTCCGCAGCTCTGTGAAG-3′	5′ FAM- 3′ BHQ-1
*GAPDH*	Probe: 5′- CCGTTGACTCCGACCTTCACCTTCC-3′ Forward primer: 5′- AGCCACATCGCTCAGACACC-3′ Reverse primer: 5′- TGACCAGGCGCCCAATACG-3′	5′HEX- 3′TAMRA

The primers and probes of the target genes and the endogenous control (GAPDH) were labelled with different fluorescent reporter dyes at the 5′ end and quencher dyes at the 3′ end and this allowed the target genes to be amplified in the same tube in a duplex qPCR reaction.

After optimizing the primer and probe PCR conditions, a duplex qPCR was performed in a 25 μl reaction volume that contained 0.3 μM forward and reverse primers of the target genes, 0.2 μM of the target probes, 0.1 μM forward and reverse primers of the *GAPDH*, 0.2 μM of the *GAPDH* probe and 2.5 μl of 1:10 dilution of the cDNA samples. The qPCR cycling conditions were as follows: 95 °C for 10 min for the first cycle (initial denaturation), 95 °C for 15 s for 40 cycles (denaturation) and 60 °C for 60 s for 40 cycles (annealing/extension). The qPCR reaction products were analyzed using Bio-Rad CFX 96 manager software (Bio-Rad, USA). The relative quantification of the target genes was calculated using the comparative CT method. The relative quantities of *JAK1*, *STAT1* and *OAS3* after normalization to the endogenous control (*GAPDH*) was given by 2 ^-ΔΔCT^ as previously described [17].

### Western blotting

To examine the effects of AFB_1_ on the protein accumulation of STAT1, the cultured HepG2 cells were treated with AFB_1_ and simultaneously stimulated with the rIFN-α for 24 h as described above. The cells were later harvested and lysed to extract total proteins using cold Radioimmunoprecipitation assay (RIPA) buffer (Thermo Scientific, Germany) containing freshly added protease and phosphatase inhibitor cocktails and ethylenediaminetetraacetic acid (EDTA) (Thermo Scientific, Germany) following a standard protocol. Aliquots of the protein samples were mixed with 2X sample buffer containing 2-beta mercaptoethanol and the mixture was heated for 5 min at 95 °C to denature the proteins. The proteins were separated by 10% sodium dodecyl sulfate polyacrylamide gel electrophoresis (SDS-PAGE) and then blotted onto 0.45 μM pore size polyvinylidene difluoride (PVDF) membranes (Bio-Rad Laboratories). The PVDF membranes were blocked in 5% non-fat dried milk in 1X Tris buffered saline with Tween-20 (TBST) before being incubated separately with STAT1 and GAPDH primary antibodies. Primary antibodies were used at the following dilutions: STAT1 (1:1000 dilution) and GAPDH (1:2500 dilution). The membranes were then incubated with the primary antibodies at 4 °C overnight. The secondary antibodies conjugated to horseradish peroxidase (Thermo Scientific, USA) were used at a dilution of 1:5000. The membranes were probed with the secondary antibody at room temperature with gentle shaking for 1 h after which bands were visualized by performing enhanced chemiluminescence using Pierce enhanced chemiluminescence (ECL) western blotting substrate (Thermo Scientific, USA). The images were captured with C-DIGIT blot scanner (Li-COR Bioscience, USA) and analyzed using image J software.

## Results

### Cytotoxic effects of AFB_1_ and IFN concentration course

**Fig. 1:**
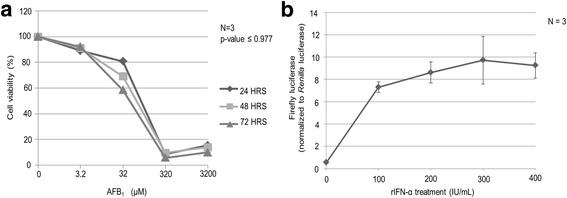
Establishing the maximum non-toxic concentration of AFB_1_ and maximum inducible concentration of rIFN-α (**a**) HepG2 cells were cultured at density of 5 × 10^4^ cells per well of the 96-well plate until they reached 60% confluence. The cells were then treated with or without increasing amount of AFB_1_ (0–3200 μM) for 24 h after which the AFB_1_ containing media were replaced with fresh media. Cytotoxicity was evaluated by MTS-based assay at 24, 48 and 72 h. The viability of cells was calculated as ratio between AFB_1_ treated cells and non-treated cells. Data are presented as mean and standard deviation of three independent experiments each performed in duplicate wells, *p*-value ≤ 0.977 as determined by one-way ANOVA. **b** Establishing the maximal IFN-α induction of ISRE driven luciferase reporter gene activity. HepG2 cells were cultured at density of 5 × 10^4^ cells per well of the 96-well plate until they reached 80% confluence. The cells were transiently co-transfected with pISRE-luc (500 ng) and pRLSV40 (1 ng). At 24 h post-transfection, the cells were treated with increasing concentration of rIFN-α. Luciferase activity was measured 24 h later. The data are presented as mean and the standard deviation of three independent experiments each conducted in duplicate wells. There was no significant difference in pISRE-luc activity of cells treated with 300 and 400 IU/ml of rIFN-α (*p*-value ≤ 0.7527)

The AFB_1_ is an extremely toxic compound which could not have been used directly on the cells. Therefore, the concentration of AFB_1_ that was not toxic to the HepG2 cells was determined using MTS based assay as described above. As shown in Fig. 1a, AFB_1_ killed the HepG2 cells in a dose-dependent fashion after exposure to concentrations up to 3200 μM for 24 h followed by maintenance of cells in AFB_1_ free media for 24, 48 and 72 h. The experiment established up to 10 μM of AFB_1_ was not toxic to the cells and was therefore used in subsequent experiments. Next the concentration of rIFN-α required to induce maximal activity of the type I IFN response pathway was determined. It was observed that the pISRE-luc activity (a measure of the type I interferon activity) increased with increasing concentration of rIFN-α and peaked at concentrations of 200-400 IU/ml (Fig. 1b).

### AFB_1_ suppresses IFN-α induced ISRE signalling

To measure the effect of AFB_1_ on the anti-cancer activity of the type I IFN response pathway, a luciferase reporter gene expressing pISRE-luc was employed. The pathway activation was induced with rIFN-α (400 IU/ml) through the transactivation of interferon stimulated response elements (ISRE). It is already established that the pathway is activated following the binding of IFN-α to the IFN-α receptors R1 and R2 on cells to trigger cascades of events leading to the transactivation of the ISRE as reviewed in Randall and Goodbourn [18].

**Fig. 2:**
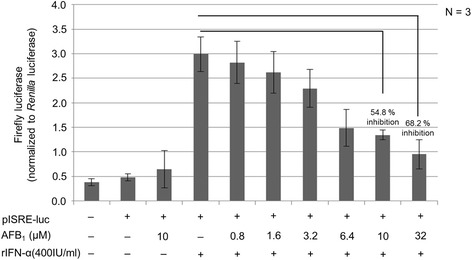
AFB_1_ inhibits IFN-α induced ISRE signalling in a dose dependent fashion. HepG2 cells were cultured at density of 5 × 10^4^ cells per well of the 96-well plate until they reached 80% confluence. The cells were transiently co-transfected with pISRE-luc (500 ng) and pRLSV40 (1 ng). The pRL40-luc which constitutively expresses the *Renilla* luciferase was included as internal control to which pISRE-luc activity was normalized. At 24 h post-transfection, the cells were stimulated with or without rIFN-α and simultaneously treated with or without AFB_1_. Transfected cells which were stimulated with rIFN-α but not treated with AFB_1_ were calculated to have 100% pISRE-luc activity. The data are presented as mean normalized pISRE-luc activity and the standard deviation of three independent experiments each conducted in duplicate wells. There was a significant difference in pISRE-luc activity of cells stimulated with rIFN-α alone compared to cells stimulated with rIFN-α and simultaneously treated with 10 μM of AFB_1_ (*p*-value ≤ 0.047)

The cultured HepG2 cells were stimulated with rIFN-α and simultaneously treated with AFB_1_ as described above. As shown in Fig. 2, the activated pathway peaked at about five-fold above background or basal levels (see fourth bar; Fig. 2). It was noted that AFB_1_ did not influence the pathway activity in anyway because cells treated with AFB_1_ alone showed same background level of pathway activity as those cells in which the pathway was not activated; the so called ‘none treated cells’ (see first bar; Fig. 2).

The addition of AFB_1_ to the cells in which the type I IFN response pathway was activated saw AFB_1_ dose dependent inhibition of the pathway activity up to 54.8% in cells treated with 10 μM of AFB_1_ and 68.2% in cells treated with 32 μM respectively (Fig. 2). The pathway activity was measured by the firefly luciferase pISRE-luc activities (Fig. 2).

The concentration of AFB_1_ and rIFN-α used in the experiment were chosen based on the following: (i) at 10 μM of AFB_1_ ≥ 90% of the cells survived (Fig. 1a), (ii) between 200 and 400 IU/ml of rIFN-α induced the maximum activity of IFN-α inducible ISRE promoter i.e. a measure of the type I IFN response pathway (Fig. 1b). In the subsequent experiments, 10 μM of AFB_1_ was used and that concentration was selected based on the fact it had the capacity to significantly inhibit the type I IFN induced signalling in HepG2 cells as measured by the firefly luciferase pISRE-luc activities (*p*-value ≤ 0.047) (Fig. 2).

### AFB_1_ inhibits transcripts expression of JAK1, STAT1 and OAS3 genes

Having demonstrated at the luciferase reporter gene assay level that AFB_1_ inhibits the type I IFN response signalling pathway, the next task was to test our hypothesis that AFB_1_ would inhibit the transcripts of key signalling elements of the pathway. The JAK-STAT-ISRE arm of the type I IFN response pathway was chosen for the study because when activated it leads to the activation of interferon responsive genes such as OAS3 whose inhibition by AFB_1_ was hypothesized in the current study.

RT-qPCR analysis of the transcripts levels of JAK1, STAT1 and OAS3 genes in the cultured HepG2 cells stimulated with or without rIFN-α (400 IU/ml) and simultaneously treated with or without AFB_1_ (10 μM) was performed.

The cells in which the pathway was activated showed about 3-fold increase in the transcripts levels of JAK1 compared to background or basal levels. However, when AFB_1_ was added to the cells in which the pathway was activated the transcripts levels of JAK1 reduced to almost half (49.1%, *p*-value ≤ 0.0001).

**Fig. 3:**
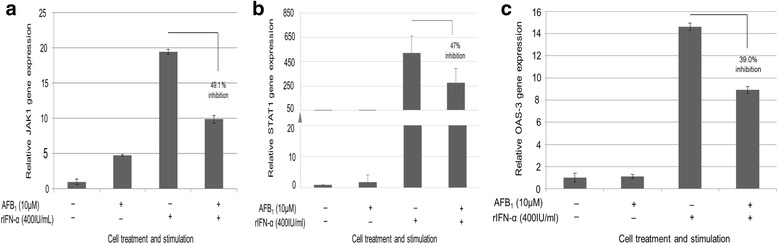
AFB_1_ inhibits the mRNA expression levels of the JAK1 (**a**), STAT1 (**b**) and OAS3. **c**. HepG2 cells were cultured at density of 5 × 10^4^ cells per well of the 6-well plate until they reached 80% confluence. The cells were stimulated with or without rIFN-α (400 IU/ml) and simultaneously treated with or without AFB_1_ (10 μM) for 24 h. Total RNA was extracted and reverse transcribed to cDNA using random primers. Quantitative PCR was performed with gene specific primers and probes for *JAK1*, *STAT1 and OAS3* respectively. The relative levels of *JAK1*, *STAT1 and OAS3* after normalization to GAPDH (endogenous control) was plotted. The relative levels of *JAK1*, *STAT1 and OAS3* were calculated using the 2^-∆∆Ct^ (Livak) method. These results are presented as mean and standard deviations of three independent experiments each performed in triplicate wells; *JAK1* (*p*-value ≤ 0.0001), *STAT1* (*p*-value ≤ 0.03) and *OAS3* (*p*-value ≤ 0.05)

Although the pathway activities in those experiments to assess the effect of *STAT1* and *OAS3* transcripts levels showed over 10-fold above background levels, similar pattern of results were seen for *STAT1* and *OAS3* because the transcripts levels were reduced by AFB_1_ to 47% (*p*-value ≤ 0.03) and 39% (*p*-value ≤ 0.05) respectively (Fig. 3b & c).

Taken together, it was observed that AFB_1_ significantly inhibited the mRNA expression levels of JAK1, STAT1 and OAS3 genes (Fig. 3). To be sure that HepG2 cells were responding to rIFN-α treatment and that the ISRE was functioning, a parallel experiment in which HepG2 cells were transiently transfected with pISRE-luc and pRLSV40-luc as described in Fig. 2 was conducted. At the time of harvest of the cells for RT-qPCR, aliquots of cell lysates from the parallel experiments were assayed for dual luciferase activity and the inhibition of ISRE activity was confirmed as described in Fig. 2 (data not shown).

### AFB_1_ inhibits STAT1 protein synthesis

Different post-transcriptional events could be involved in translating mRNAs into proteins [19] suggesting that lower mRNA levels might not necessarily corresponds to lower protein expression and vice versa. Therefore, western blot assay was employed to ascertain whether the inhibition of the mRNA expression level of STAT1 by AFB_1_ would ultimately affect its translation into proteins as well (see Fig. 3).

Again the cultured HepG2 cells were stimulated and treated as described in Fig. 3. Protein extracts of HepG2 cells stimulated with or without rIFN-α and treated with AFB_1_ (10 μM) were analysed by western blotting for STAT1 using Glyceraldehyde 3-phosphate dehydrogenase (GAPDH) as a loading control. Briefly, cell lysates were prepared and thereafter, protein samples were separated on SDS-PAGE and immunoblotted with anti STAT1 and anti-GAPDH antibodies respectively. In order to ensure that the cells were functionally responding to stimulation and treatment at the time when the lysates were prepared, a parallel experiment in which the cells were transiently transfected with pISRE-luc and pRLSV40 as described in Fig. 3 were assayed for dual luciferase activity and the inhibition of the type I IFN response pathway activity by AFB_1_ was confirmed (data not shown).

**Fig. 4:**
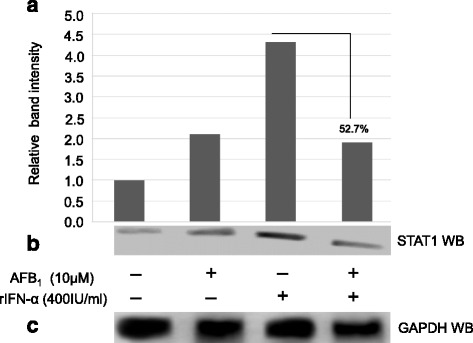
AFB_1_ inhibits STAT1 protein synthesis. HepG2 cells were cultured at density of 5 × 10^4^ cells per well of the 6-well plate until they reached 80% confluence. The cells were stimulated and treated as described above. After blotting, the membranes were immunoblotted with antibody against the STAT1 epitope of the STAT1 protein as primary antibody followed by horseradish peroxidase conjugated goat anti-rabbit antibody as secondary antibody. The membranes were also probed for GAPDH which was used as endogenous control. The dilutions of the primary antibodies used against STAT1 and GAPDH proteins were as follows: STAT1 (Thermo Scientific, cat no PA5-34504; 1:1000), GAPDH (Thermo Scientific, cat no QE212271; 1:2500). The secondary antibody used was polyclonal goat anti-Rabbit conjugated to HRP (Thermo Scientific, cat no PI31460, 1: 5000). The blotted membranes were developed using enhanced chemiluminescence reagents and the protein bands were detected and captured with C-DIGIT blot scanner imaging device. (**a**) The relative STAT1 protein intensity was quantified using Image J software package. (**b**) Western blot analysis of STAT1 protein. (**c**) Western blot analysis of GAPDH protein as loading control

Consistent with the results shown in Fig. 3, STAT1 accumulation peaked in cells in which the type I IFN pathway was activated in the absence of AFB_1_ (see Fig. 4; band 2 from right). However, on the contrary the STAT1 accumulation was substantially reduced in cells in which the pathway was activated in the presence of AFB_1_ (see Fig. 4; band 1 from right). Again, cells in which the type I IFN pathway was not activated showed low background levels of STAT1 presence (see Fig. 4; bands 1 and 2 from left). Also consistent with Fig. 3 the protein levels of GAPDH were not affected by AFB_1_ as their levels were shown to be equal in all cells regardless of the AFB_1_ treatment or stimulation with rIFN-α.

## Discussion

AFB_1_ is a potent hepatocarcinogen [3, 4] which when ingested is metabolized by CYP450 class of enzymes in the liver to AFB_1_, 8-9 exo epoxide [3, 4]. The AFB_1_ 8-9 exo epoxide binds DNA and induces AGG to AGT transversion mutation at codon 249 in the tumour suppressor gene p53 [20, 21]. This mutation leads to the inhibition of the p53 mediated transcription and underlines the mechanism by which AFB_1_ causes HCC [22]. It must be stated however that, the mechanism by which AFB_1_ causes HCC may not be limited to p53 mutation alone and that AFB_1_ may also induce cancers by deregulating other anticancer signalling pathways. For example Ubagai et al. [23] demonstrated that AFB_1_ may also induce tumourigenesis by deregulating the insulin-like growth factor 1 receptor (IGF-IR) signalling pathway suggesting that AFB_1_ may also induce tumourigenesis by deregulating other anticancer pathways such as the type I IFN signalling pathway. In this study we tested the hypothesis that AFB_1_ would suppress/inhibit the type I IFN signalling pathway and thus provide another mechanism by which AFB_1_ may cause cancer. Findings from the current study in which AFB_1_ was shown to inhibit the type 1 IFN response pathway by targeting the key signalling elements are consistent with that of Jiang et al. [24] who reported that AFB_1_ inhibited the mRNA expression levels of IL-2, IL-4, IL-6, IL-10, IL-17, IFN-γ and TNF-α in the small intestines of broilers treated with AFB_1_. Moreover, AFB_1_ has also been reported to inhibit mRNA and protein expression levels of IL-4, IL-6, IL-10 in the peritoneal macrophages and splenic lymphocyte cell lines [25, 26] and STAT5A gene mRNA expression levels in bovine mammary epithelial cells [27]. One way by which the type I IFN signalling response exerts its anti-cancer and antiviral response is through the activation of the JAK-STAT-ISRE arm of the pathway. One component of the JAK-STAT-ISRE signalling pathway considered to have tumour suppressor function is STAT1 [28]. When activated, STAT1 suppresses tumour development by inducing apoptosis [29] and also inhibit tumour angiogenesis [30]. Therefore the suppression of STAT1 by AFB_1_ at the both transcription and translational levels as demonstrated in the current study will impair the ability of STAT1 in orchestrating the expression of myriad of genes which are required to promote apoptosis, inhibit cell proliferation and angiogenesis in response to AFB_1_.

**Fig. 5:**
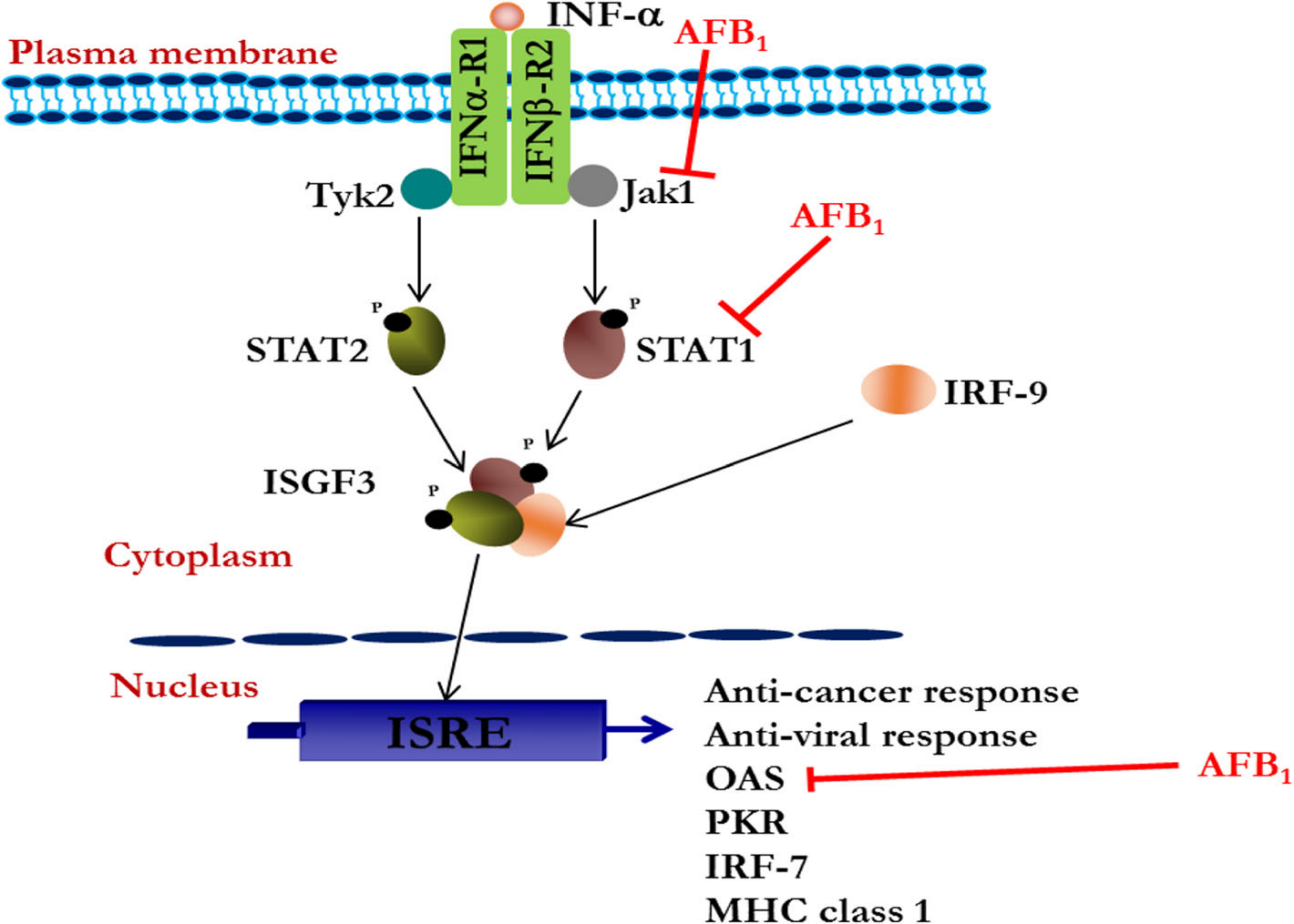
Proposed deregulation of type I IFN signalling pathway by AFB_1_. The type I IFN signalling pathway begins when ligand such as IFN-α/β molecules binds to IFNAR1/2 which are associated with Tyk2 and JAK1 respectively. The interaction between IFN-α/β and the receptor results in phosphorylation and activation of Tyk2 and JAK1. The activated JAKs in turn recruit and phosphorylate STAT1 and STAT2 on tyrosine 701 (727 as well) and 690 respectively. The phosphorylated and activated STAT1 and STAT2 come together and form heterodimer. The dimerized STAT1 and STAT2 recruits IRF-9 and form the ISGF-3 transcription factor complex. The ISGF-3 enters the nucleus and interacts with ISRE which results in the transcription of IFN-inducible genes that switch on the anti-cancer as well as the anti-viral defense system. The current study has demonstrated that AFB_1_ suppresses or inhibits the mRNA expression levels of JAK1, STAT1 and OAS3 (indicated by red font and straight lines crossed at one end). In addition, protein expression level of STAT1 was also demonstrated to be inhibited by AFB_1_

The activation of the JAK-STAT-ISRE arm of the type I IFN signalling pathway results in the activation of the so called interferon responsive elements including the OAS3, PKR, Mx etc as reviewed in Randall and Goodbourn [18]. The OAS3 pathway when activated leads to the establishment of antiviral state by inhibiting protein synthesis which culminates in the destruction of both viruses and infected cells [31, 32]. The OAS3 has also been reported to play a role in inhibiting tumour development by inducing apoptosis and anti-proliferative responses [33, 34]. The activation of the JAK-STAT-ISRE signalling pathway starts upon the phosphorylation and activation of JAK1 and TYK2 when the correct ligands bind to the IFNAR (Fig. 5). The activated JAK1 and TYK2 in turn phosphorylate STAT1 and STAT2 setting in motion a cascade of events which finally initiates the transcription of IFN-inducible genes switching on the anti-cancer and anti-viral effects as reviewed in Randall and Goodbourn [18]. Therefore, any stimulus which deregulates the expression of either JAK1 or TYK2 could potentially deregulate the entire type I IFN response signalling pathway and thereby weaken the immune system and thus predispose individuals to infections and or cancer.

Taken together, the current study has revealed for the first time the inhibition of the type I IFN response pathway by AFB_1_ via the inhibition of the transcripts of *JAK1*, *STAT1* and *OAS3* and also inhibit the protein accumulation of STAT1. The findings of the current study could be another mechanism by which AFB_1_ may cause HCC (Fig. 5).

## Conclusions

In conclusion the current study has shown that AFB_1_ down-regulated the type I IFN response pathway by significantly inhibiting the key signalling elements such as *JAK1*, *STAT1 and OAS3* and also the STAT1 protein. Findings from this study reveals the negative effects of AFB_1_ on the health of people who consume AFB_1_ contaminated food as evidenced by its ability to inhibit and or deregulate the innate immune response. In view of this it is recommended that public education is intensified in Ghana and other developing nations of the world where AFB_1_ contaminated food form a greater portion of the diets so as to reduce HCC and its associated deaths.

## Abbreviations

AFB_1_: Aflatoxin B_1_
AIDS: Acquired immunodeficiency syndrome
CYP450: Cytochrome p450
DMEM: Dulbecco’s modified eagles’ medium
EDTA: Ethylenediaminetetraacetic acid
FBS: Foetal bovine serum
GAPDH: Glyceraldehyde 3-phosphate dehydrogenase
HBV: Hepatitis B virus
HCC: Hepatocellular carcinoma
HCV: Hepatitis C virus
HEPES: (4-(2-hydroxyethyl)-1-piperazineethanesulfonic acid
IARC: International agency for research on cancer
IFN: Interferon
IFN-α: Interferon-alpha
IFN-β: Interferon beta
IGF-IR: Insulin-like growth factor 1 receptor
IL: Interleukin
IRF-9: Interferon regulatory factor 9
ISGF-3: Interferon stimulated gene factor 3
JAK1: Janus activated kinase 1
MEM: Minimum essential medium
OAS3: Oligo adenylate synthetase 3
PBL: Peska Biomedical Laboratories
ppb: Part per billion
PVDF: Polyvinylidene difluoride
SDS-PAGE: Sodium dodecyl sulfate polyacrylamide gel electrophoresis
STAT1: Signal transducer and activator of transcription 1
TNF-α: Tumour necrosis factor-alpha
